# An Injectable, Self-Healing Hydrogel Based on G-Quadruplexes/Phenylboronic Acid Composites with Antibacterial Activity

**DOI:** 10.3390/gels12070612

**Published:** 2026-07-09

**Authors:** Hongyi Yang, Hui Jiang

**Affiliations:** 1 School of Pharmacy, Nanjing University of Chinese Medicine, Nanjing 210023, China; 13605213079@163.com; 2 School of Biological Science and Medical Engineering, Southeast University, Nanjing 210096, China

**Keywords:** injectable hydrogel, self-healing, G-quadruplex, dynamic boronate ester, supramolecular, antibacterial activity

## Abstract

Injectable and self-healing hydrogels hold tremendous promise for biomedical applications; however, synchronously integrating robust mechanical adaptability, excellent cytocompatibility, and intrinsic antibacterial capabilities within a single matrix remains a significant challenge. In this study, we engineered an injectable, self-healing hydrogel based on dynamic cross-linking using guanosine-derived G-quadruplex supramolecular self-assembly and 3-aminophenylboronic acid (3-APBA)-mediated dynamic boronate ester. Systematic evaluation of various phenylboronic acid derivatives, GMP concentrations, K^+^ sources, and 3-APBA levels on gelation behavior yielded an optimized formulation. Scanning electron microscopy revealed that the optimized hydrogel exhibits a continuous, interconnected porous network structure after lyophilization. Thioflavin T fluorescence enhancement assays and circular dichroism spectroscopy further verify the formation of G-quadruplex-related ordered assemblies within the system. Rheological assessments demonstrate elasticity-dominated gel behavior, pronounced shear-thinning characteristics, and reversible structural breakdown and recovery under high and low strain cycles, indicating excellent injectability and self-healing properties. In vitro cytocompatibility evaluations show that the hydrogel possesses favorable cellular compatibility. Further antimicrobial studies reveal excellent in vitro antibacterial activity against *Staphylococcus aureus* and *Escherichia coli*. In summary, the injectable, self-healing G-quadruplex hydrogel constructed in this study integrates a porous architecture, dynamic reversibility, and robust biological functionality, highlighting its promising potential in antibacterial applications.

## 1. Introduction

Injectable and self-healing hydrogels have attracted tremendous attention in the fields of biomedicine and soft materials [1] due to their excellent structural adaptability and dynamic responsiveness [2,3]. Compared with traditional pre-formed hydrogels, injectable hydrogels can be administered to deep or irregular tissue defects via minimally invasive methods (e.g., syringe injection) and rapidly form gels in situ after extrusion to effectively match complex morphologies [4,5]. Furthermore, their self-healing property allows hydrogels to autonomously recover their structural and network integrity after external mechanical damage [6], which is crucial for maintaining the stability of the materials in dynamic physiological environments [7,8]. Therefore, the development of multifunctional dynamic hydrogels integrating injectability, rapid self-healing ability, and additional bioactivities (such as antibacterial properties) has become a key driving force in materials science [9].

Although injectable and self-healing hydrogels exhibit significant advantages in adapting to complex morphologies and resisting mechanical disruption, their practical biomedical applications are often severely restricted by the risk of bacterial infection [10,11]. In scenarios such as local filling, minimally invasive delivery, or dynamic biointerface adaptation, the highly hydrated and porous three-dimensional networks of hydrogels can easily become breeding grounds for bacterial colonization, thereby destroying the structural integrity of the material and triggering localized inflammation [12]. Traditional antibacterial strategies usually rely on the physical doping of exogenous antibiotics or metallic silver/copper nanoparticles. However, these physical encapsulation systems frequently suffer from the uncontrolled burst release of therapeutic agents and the risk of long-term cytotoxicity, failing to provide sustained infection control [13]. Furthermore, the direct incorporation of exogenous antimicrobial agents often perturbs or even disrupts the intrinsic dynamic cross-linking networks of hydrogels, severely compromising their mechanical properties and self-healing efficiency. In contrast, hydrogels directly assembled from building blocks with inherent antimicrobial activity represent a safer and more sustainable alternative [14,15]. Therefore, developing a multifunctional supramolecular hydrogel that effectively integrates robust self-assembly motifs, biocompatibility, and intrinsic antibacterial properties remains a major challenge [16].

Biomolecules such as peptides and nucleosides are highly favored in the biomaterials sector due to their inherent biocompatibility, bioactivity, and cost-effectiveness. Guanosine, a ubiquitous nucleoside and a fundamental constituent of nucleic acids [17], boasts a broad spectrum of biological applications. Among its derivatives, guanosine hydrogels represent a class of smart soft materials formed via the supramolecular self-assembly of guanosine or its analogs [18]. The core driving force behind their ability to form diverse self-assembled structures is the intermolecular hydrogen bonding of guanosine molecules into G-quartet structures. Stabilized by alkali metal cations (particularly K^+^) [19], these quartets further assemble into nanofibers via π-π stacking, ultimately entangling to form a three-dimensional macroscopic network [12]. Because the cross-linking nodes driven by non-covalent interactions (such as hydrogen bonds) are relatively weak, the network structure is readily disrupted under external mechanical force, conferring characteristic shear-thinning properties [20]. Conversely, upon the removal of external force, these intermolecular non-covalent interactions are rapidly re-established, reforming the G-quartet structures and restoring the network’s shape and integrity, thereby endowing the material with self-healing capabilities. Despite the significance of such guanosine hydrogels, their primary obstacles in biomedical applications remain their poor long-term stability and the requirement for excessive cations [12]. Therefore, introducing dynamic covalent bonds via chemical modification to construct a double-network structure can significantly enhance the performance and controllability of guanosine hydrogels. Studies have revealed that boronate ester bonds also exhibit a degree of reversibility (hydrolysis and reformation) under physiological conditions [21,22], which further augments the dynamism and self-healing capacity of the network [23]. Concurrently, the introduction of this covalent cross-linking can dramatically improve the mechanical strength of the gel while preserving its shear-thinning nature, making it highly suitable for product stability requirements. The synergistic combination of these two types of interactions holds the promise of endowing materials with both structural stability and dynamic responsiveness, rendering them exceptionally well-suited for biomedical applications [24].

In this study, we have engineered an injectable supramolecular hydrogel based on dynamic cross-linking between G-quadruplex and 3-aminophenylboronic acid (3-APBA). Unlike conventional phenylboronic acid (PBA) and its derivatives (e.g., mercapto-PBA) that typically form fragile networks or undergo rapid matrix collapse under physiological conditions, we strategically selected 3-APBA as a distinct functional cross-linker [25]. The critical uniqueness of 3-APBA stems from its amino group, which facilitates extensive intermolecular hydrogen bonding and electrostatic interactions. This structural feature effectively prevents network collapse and endows the hydrogel scaffold with remarkable stability [26]. We systematically investigated the influence of GMP concentration, K^+^ source, and 3-APBA concentration on gel formation. The as-obtained hydrogel presents a continuous, interconnected porous microstructure. Crucially, circular dichroism (CD) spectroscopy combined with in situ ThT fluorescence enhancement assays firmly corroborate the characteristic formation of ordered G-quadruplex architectures within the dynamic matrix [27,28]. Simultaneously, the hydrogel demonstrates superior rheological properties, encompassing elasticity-dominated gel behavior, strain-triggered reversible breakdown and recovery, and shear-thinning responses essential for injectability. Furthermore, the hydrogel exhibits robust self-healing behavior, excellent cytocompatibility, and antibacterial activity under the tested in vitro conditions, underscoring its immense potential for flexible biomedical scenarios, such as wound dressing material. By establishing the correlation between formulation parameters, microstructure, and macroscopic performance, this research aims to provide a viable strategy for the construction of dynamic supramolecular hydrogels with promising translational potential.

## 2. Results and Discussion

### 2.1. Preparation and Optimization of Guanosine Derivative–Phenylboronic Acid Hydrogels

**Figure 1:**
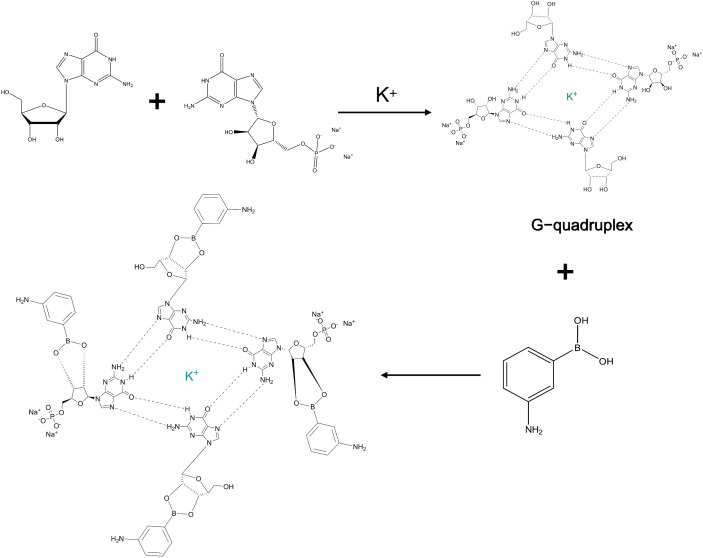
Schematic illustration of the hydrogel formation mechanism. Schematic illustration of hydrogel preparation.

**Figure 2:**
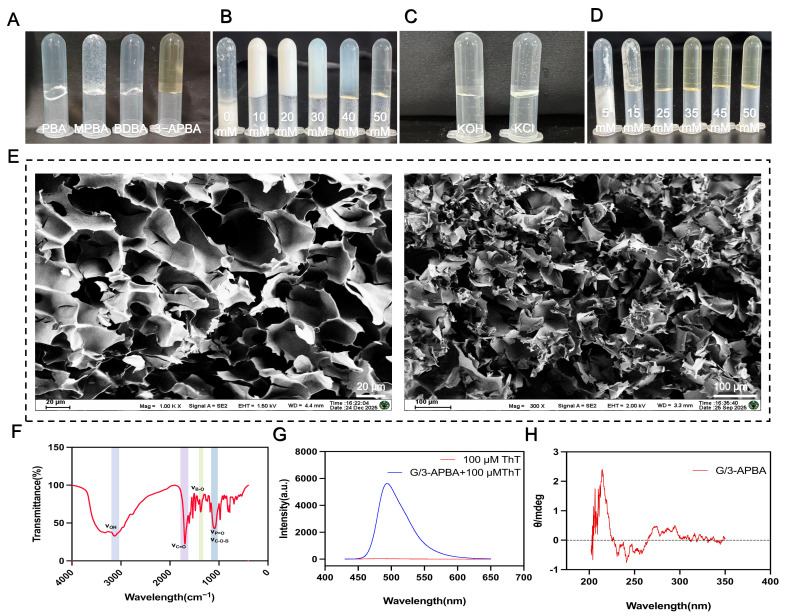
Preparation, screening, and morphological and structural characterization of G/3-APBA hydrogels. (**A**) Visual comparison of hydrogels formed with PBA, MPBA, BDBA, and 3-APBA. (**B**) Comparison of hydrogels incorporated with 0, 10, 20, 30, 40, and 50 mM of GMP (from left to right). (**C**) Comparison of hydrogels formed in KOH and KCl systems. (**D**) Comparison of hydrogels comprising different concentrations of 3-APBA (5, 15, 25, 35, 45, and 50 mM) (from left to right). (**E**) SEM images of the hydrogel. (**F**) FTIR spectrum of the G/3-APBA hydrogel. (**G**) Fluorescence intensity of G/3-APBA +100 μM of ThT and 100 μM of free ThT. (**H**) CD spectrum of the G/3-APBA hydrogel.

The formation process of guanosine derivative–phenylboronic acid (3-APBA) hydrogels is illustrated in Figure 1. Mechanistically, the hydrogels are assembled via a synergistic “dual-dynamic” network with distinct structural compartmentalization. Specifically, potassium-stabilized G-quadruplex supramolecular assemblies function as the rigid skeletal framework, conferring structural rigidity and characteristic shear-thinning properties. Simultaneously, dynamic boronate ester bonds serve as reversible elastic cross-links, ensuring optimal cross-linking density and rapid self-healing resilience [12]. The strategic incorporation of 3-APBA is driven by its amino group, which orchestrates extensive intermolecular hydrogen bonding and electrostatic interactions. Under physiological conditions, these non-covalent forces synergistically deliver robust electrostatic stabilization for the negatively charged tetrahedral boronate ester intermediates. This cooperative integration effectively prevents matrix collapse, affording the multicomponent system superior structural integrity [26]. To identify the most stable gel system, we first compared the impact of PBA derivatives modified with different functional groups on covalent cross-linking capacity. As shown in Figure 2A, when guanosine was combined with PBA, 4-mercaptophenylboronic acid (MPBA), 1,4-benzenediboronic acid (BDBA), and 3-APBA in a K^+^-containing system, the gel formed by the 3-APBA group maintained its original gel morphology even after long-term inversion (2 weeks), indicating that the 3-APBA composite gel system is significantly more stable. The superior stability of the 3-APBA composite gel system can likely be attributed to the electrostatic interaction between the electron-deficient boron atom in the phenylboronic acid and the electron-rich nitrogen atom in the amino group [29].

Guanosine monophosphate (GMP) is a low-molecular-weight gelator. Compared to other nucleobases, guanine possesses more hydrogen-bonding sites, thereby enabling the formation of diverse supramolecular aggregates such as G-quadruplexes, dimers, ribbons, and sheets [30]. Consequently, we investigated the effect of incorporating varying concentrations of GMP on gel formation. As the GMP concentration increased, the gelation stability of the system progressively improved, with the 50 mM GMP group exhibiting the optimal macroscopic gel state (Figure 2B). This result indicates that an appropriate amount of GMP facilitates enhanced G-quadruplex-related assembly and provides a more stable supramolecular skeleton for the three-dimensional network [19].

The G-quadruplex units formed by guanosine and its derivatives are typically stabilized by central metal ions, where cations are intercalated between two G-quartet planes to form a “sandwich” binding mode via cation–dipole interactions. It is well established that among the monovalent cations (e.g., Na^+^, K^+^, Rb^+^, and NH_4_^+^) and certain divalent cations (including Sr^2+^, Ba^2+^, and Pb^2+^) capable of stabilizing these structures [31], K^+^ is the most effective cation for G-quadruplex stabilization [32]. We further explored the influence of K^+^ sources on guanosine-based assembly. Compared to KCl supplementation, the system containing 25 mM of KOH presented a superior gelation state (Figure 2C). This is because KOH, acting as a highly efficient reagent, can simultaneously satisfy the requirements of both dynamic networks. Specifically, K^+^, as the most effective cation, stabilizes the G-quadruplex core through optimal cation–dipole interactions; meanwhile, compared with Cl^−^, OH^−^ provides the mild alkaline environment required for the deprotonation of boronic acid. When the pH value of the medium is greater than the p*K*_a_ value of the boronic acid, the equilibrium shifts toward the formation of boronate esters, and the negatively charged tetrahedral boronate esters are more stable in near-alkaline aqueous solutions [12].

Finally, the concentration effects of 3-APBA on hydrogel stability were compared by using 3-APBA at a content ranging from 5 to 50 mM (Figure 2D). The 35 mM 3-APBA group maintained a highly stable gel state both immediately after preparation and after one week of storage at room temperature, demonstrating that the dynamic boronate ester cross-linking density and the network rearrangement capacity reached an optimal balance at this concentration.

The gel formulation comprising precursors of 50 mM of guanosine, 25 mM of K^+^ (via KOH), 50 mM of GMP, and 35 mM of 3-APBA was consequently determined to be optimal. Crucially, this facile, purification-free one-pot preparation protocol exhibits exceptional batch-to-batch reproducibility. Across multiple independently prepared sample cohorts, this approach consistently yields identical macroscopic gelation behavior and highly uniform rheological moduli.

### 2.2. Morphological and Structural Characterization of G/3-APBA Supramolecular Hydrogels

The morphology and porosity of hydrogels are of paramount importance for cellular proliferation and adhesion. Microscale pores facilitate uniform cell distribution, and optimal pore sizes enable efficient nutrient transport, metabolic exchange, and oxygen diffusion, whereby a porous architecture promotes cell proliferation and infiltration within the gel scaffold [33]. Notably, smaller pore sizes (80–120 μm) play a positive role during the inflammatory phase [34].

In this study, the micro-morphology of the lyophilized hydrogel samples was acquired using scanning electron microscopy (SEM). As shown in Figure 2E, the sample displays a distinctly interconnected porous skeleton with an abundant distribution of pores, where the pore walls are composed of wrinkled, lamellar structures. Although the porous morphology observed after lyophilization under high vacuum may not perfectly replicate the fully native hydrated state of the hydrogel network, overall, the SEM results substantially and effectively validate the considerably high interconnectivity among the pores within the system, indicating that the system can still construct a relatively intact spatial network after macroscopic gelation [35,36]. Furthermore, the microscale pore dimensions are highly conducive to exudate exchange and local microenvironmental mass transport in biomedical systems [37].

To elucidate the non-covalent and dynamic covalent interactions within the G/3-APBA hydrogel network, FTIR spectroscopic characterization was performed. Figure 2F depicts the hydrogel’s sharp and prominent characteristic absorption peak at 1694.72 cm^−1^, which is assigned to the C=O stretching vibration of the guanine group. Its distinct sharpness and position strongly suggest that the carbonyl groups in the system participate in the construction of a highly ordered Hoogsteen hydrogen-bonded network, thereby confirming the successful self-assembly of the G-quadruplex backbone within the hydrogel [31]. Furthermore, the infrared spectrum verifies the formation of dynamic covalent cross-linking within the system. The spectrum displays a clear absorption band at 1372.63 cm^−1^, which is a typical characteristic peak for B-O stretching vibrations. Concurrently, the broad and complex signal band centered at 1089.44 cm^−1^ represents the overlapping stretching vibrations of the P=O groups from the GMP backbone and the newly formed C-O-B bonds. These comprehensive spectral signatures indicate that 3-APBA reacted with the cis-diol groups on the ribose rings of the G/GMP assemblies, successfully integrating into the supramolecular network via dynamic boronate ester bonds [22]. Lastly, the spectrum presents a broad and intense absorption band near 3126.87 cm^−1^, corresponding to O-H and N-H stretching vibrations, which reflects the extensive intermolecular and intramolecular hydrogen bonding network present within the hydrogel, as well as the highly hydrated physical nature of the system [38].

Molecules rich in guanosine derivatives self-assemble into supramolecular structures via the ordered alignment of G-quadruplexes; these secondary structures are aggregated based on the Hoogsteen hydrogen bonding of guanine groups [27]. When Thioflavin T (ThT) specifically intercalates between the planar G-quartets of a G-quadruplex, its molecular conformation is locked, and rotational freedom is restricted, resulting in a hundred- to thousand-fold enhancement in fluorescence intensity. To verify the G-quadruplex assembly potential within the hydrogel, ThT was introduced to detect G4 structures via fluorescence spectroscopy (Figure 2G) [39]. The results show that while the fluorescence signal of the free ThT solution remains near the baseline, the ThT-loaded hydrogel exhibits a significantly enhanced emission peak at approximately 490–500 nm, reaching an enhanced intensity of up to 180-fold. This ThT fluorescence enhancement signifies its binding within the hydrogel network, confirming the formation of G-quadruplex-related ordered structures within the hydrogel system [28]. To further provide chiroptical evidence for the ordered guanosine-derived assembly, CD spectroscopy was performed on the optimized G/3-APBA hydrogel (Figure 2H) [40]. The CD spectrum displayed distinct cotton effects, including a pronounced positive band in the short-wavelength region and broad weak positive signals in the 270–295 nm region, accompanied by a negative band around 240–255 nm. This chiral spectroscopic profile indicates that the guanosine units are not randomly dispersed but are organized into ordered stacked aggregates within the hydrogel matrix [41]. Importantly, because CD signals of supramolecular hydrogels can be influenced by turbidity and light scattering, the CD result was interpreted together with the ThT fluorescence enhancement and FTIR analysis. The mutually supportive evidence from FTIR, ThT fluorescence, and CD spectroscopy therefore confirms the formation of G-quadruplex-related ordered supramolecular structures and their integration with dynamic boronate ester cross-links in the G/3-APBA hydrogel. Overall, the homogeneously porous three-dimensional network structured by the G/3-APBA hydrogel not only provides a structural foundation for its stable macroscopic properties but also establishes an excellent morphological basis for biological applications.

### 2.3. Rheological Evaluation of G/3-APBA

To assess the mechanical properties of the G/3-APBA hydrogels, systematic rheological characterization was conducted at a physiological temperature of 37 °C to accurately evaluate their performance for potential biomedical applications. Notably, the storage moduli (G′) of the optimized hydrogel network align well with the native biomechanical environments of soft tissues and skin secretions, ensuring excellent tissue compliance and mechanical compatibility [7]. Frequency sweeps (0.01–100 Hz; 1% strain), strain sweeps (0.1–1000%; 1 Hz), and alternating high/low strain cycles (1%/500%; three cycles) were employed to evaluate gel stability, yielding behavior, and reversible restructuring capacity. Concurrently, shear rate sweeps were conducted to determine the relationship between viscosity and shear rate, revealing shear-thinning characteristics and further supporting the material’s injectability. In the rheological analysis, G′ denotes the storage modulus, representing the elastic energy stored during deformation; G″ denotes the loss modulus, representing the energy dissipated due to viscous deformation [4].

**Figure 3:**
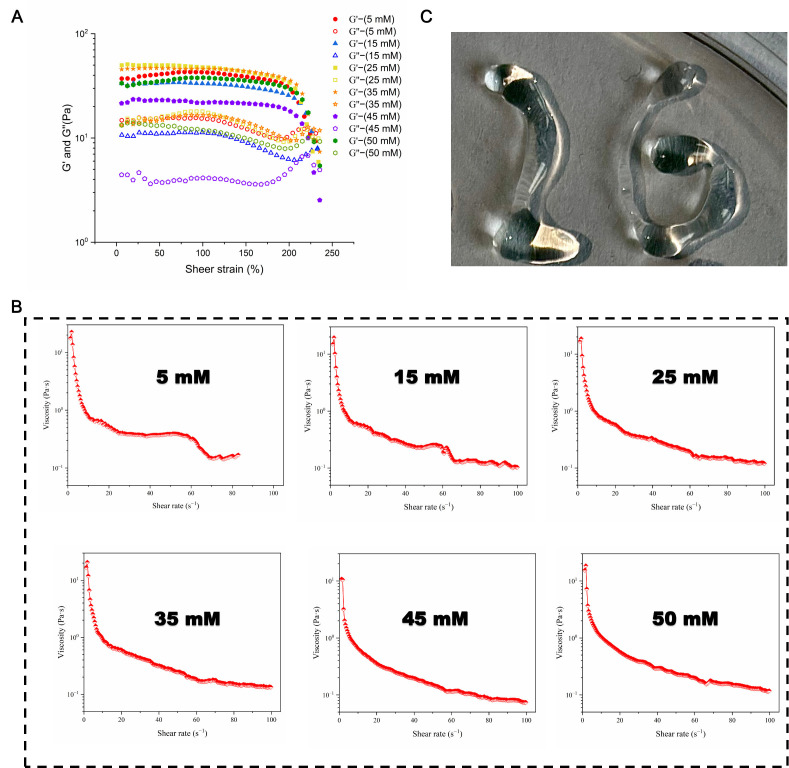
Injectability analysis of G/3-APBA hydrogels with 3-APBA concentrations of 5, 15, 25, 35, 45, and 50 mM, respectively. (**A**) Amplitude sweeps representing rheological properties. (**B**) Viscosity versus shear rate sweeps. (**C**) Macroscopic demonstration of the injectability of the hydrogel prepared with 35 mM of 3-APBA, showing smooth extrusion through a syringe and the capability to maintain stable morphology and pattern construction on a substrate.

The dynamic strain sweep profiles for samples with different 3-APBA concentrations are presented in Figure 3A. When the strain is below 250%, G′ exceeds G″ across all tested hydrogel groups, indicating that the hydrogel network structure remains stable within this regime and exhibits predominantly elastic, solid-like behavior [12]. As the strain surpasses 250%, G″ supersedes G′, denoting material yielding and a transition from solid-like to liquid-like behavior. Notably, the hydrogels made of 25 mM and 35 mM 3-APBA exhibited the highest storage moduli (G′) within the linear viscoelastic region. These findings suggest that the hydrogels possess superior structural stability under minor deformations, which is highly advantageous for maintaining structural integrity in practical applications [2].

Concurrently, all hydrogel groups exhibited pronounced shear-thinning behavior during the shear rate escalation, with viscosity decreasing significantly as the shear rate increased (Figure 3B). The viscosity of all 3-APBA concentration variants declined continuously with increasing shear rates, maintaining an overall magnitude between 10^0^ and 10^−1^ Pa·s. This characteristic shear-thinning curve (Figure 3B) unambiguously supports the injectability of the hydrogel [3]. Under high-shear conditions, the material’s viscosity decreases and its fluidity increases, which effectively reduces injection resistance and ensures continuous extrusion [42]. Upon the cessation of shear, combined with the aforementioned strain cycling results, the system can rapidly revert to the gel state via dynamic network reconfiguration, thereby achieving in situ molding and structural stabilization.

Synthesizing the results from strain sweeps and shear-thinning analyses, different 3-APBA concentrations present varying trade-offs among “network strength, yield flow, and structural recovery”. Specifically, the 35 mM group exhibited the highest initial storage modulus (G′_0_ = 42.6 Pa), far exceeding those of both the lower concentration group (15 mM and 24.1 Pa) and the higher concentration group (45 mM and 20.3 Pa). Concurrently, it displayed the broadest linear viscoelastic region (LVR), featuring the peak yield strain (γy = 175%) and the maximum flow point (γf = 222%). Despite possessing the highest mechanical rigidity, the 35 mM hydrogel maintained an exceptional third-cycle structural recovery rate of 95.3% after severe deformation. Furthermore, the macroscopic extrusion experiment confirmed that the optimized hydrogel could be smoothly extruded through a syringe and retain a stable shape after deposition (Figure 3C). Therefore, the injectability of the G/3-APBA hydrogel is supported by three complementary observations: shear-thinning behavior under increasing shear rate, structural recovery after high-strain disruption, and direct syringe extrusion with post-deposition shape retention. These results indicate that the hydrogel can flow during injection and rebuild its gel-like network after the external shear force is removed. Consequently, the hydrogel made of 35 mM 3-APBA was selected as the optimized formulation for subsequent biological evaluations.

**Figure 4:**
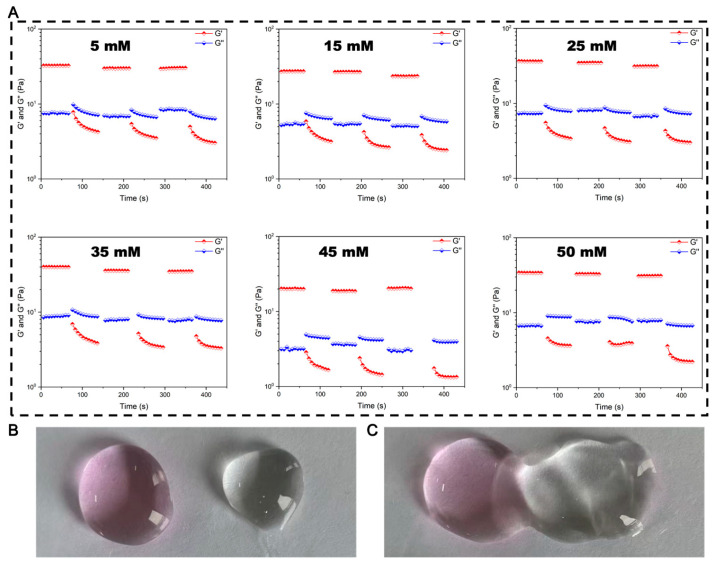
Self-healing performance analysis of G/3-APBA hydrogels. (**A**) Dynamic step-strain sweeps of hydrogels at various 3-APBA concentrations. (**B**,**C**) Macroscopic self-healing behavior of the G/3-APBA hydrogel. Two severed sections of the hydrogel are rejoined, demonstrating integration into a singular, healed whole (one segment is dyed with Rhodamine B for enhanced visualization).

To further validate the structural recovery capacity of the G/3-APBA hydrogels, alternating high/low-strain cycle tests were conducted for samples across varying 3-APBA concentrations (Figure 4A). Under high-strain conditions (500%), a sharp decline in G′ was observed for all samples, indicating that the network junctions were severely disrupted or experienced slippage under intense shear. Upon the strain returning to a low level (1%), G′ rapidly recovered to reconstruct a stable plateau. This highly reproducible “breakdown-recovery” cycle was consistently observed across three consecutive iterations. This outcome firmly establishes the system’s remarkable capacity for reversible structural reconfiguration, epitomizing classical self-healing characteristics [43].

To visually corroborate the macroscopic self-healing behavior conferred by dynamic boronate ester bonds, Rhodamine B was doped into a subset of the G/3-APBA hydrogel to enhance contrast. Subsequently, the doped hydrogel was physically bisected using external pressure. The two distinct gel segments were then brought into direct physical contact to initiate the self-healing process. As explicitly observed in Figure 4B, the interface between the differently colored hydrogel pieces seamlessly merged, and the boundary line became indistinct. This phenomenon occurs because, upon contact, the fundamental small molecules of the hydrogel rapidly bridge the interface via non-covalent interactions, effectively fusing the two segments into a single cohesive unit, visually confirming the hydrogel’s intrinsic self-healing capability. Furthermore, prolonged rheological tracking confirms that even after long-term storage at room temperature, its various rheological properties remain stable.

### 2.4. Biosafety Evaluation of G/3-APBA

**Figure 5:**
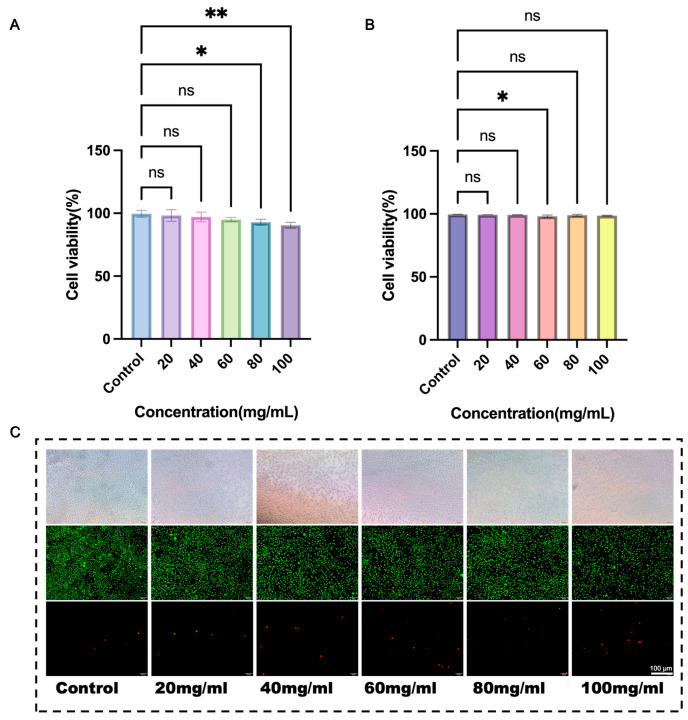
Biosafety evaluation of the hydrogels. (**A**) Cytotoxicity assessment of the G/3-APBA hydrogel against L929 fibroblasts via MTT assays. (**B**) Quantitative analysis of L929 fibroblast survival rates treated with G/3-APBA hydrogel via live/dead staining. (**C**) Representative fluorescence microscopy images of Calcein-AM/PI live (green) and dead (red) staining of L929 fibroblasts following treatment with G/3-APBA hydrogel (scale bar = 100 μm). Data are presented as mean ± SD from three independent experiments (*n* = 3, * *p* < 0.05, ** *p* < 0.01).

Biocompatibility is an absolute prerequisite for the translational application of hydrogels in biomedicine. Although guanosine, as an endogenous natural nucleoside, does not provoke severe inflammatory or immune rejection responses, the resulting phenylboronate ester polymer network structure—influenced by both cross-linking density and solid polymer concentration—may potentially hinder intercellular communication and functionality, culminating in cell death [44]. To stringently evaluate the in vitro cytocompatibility of the hydrogel system, L929 mouse fibroblasts were selected as they represent the standard ISO 10993 model for evaluating the cytotoxicity of biomaterials [45]. MTT assays were utilized to determine the relative viability of cells treated with extraction media from varying hydrogel concentrations. As depicted in Figure 5A, following a 24 h co-incubation of L929 fibroblasts with gel extracts at concentrations of 20, 40, 60, 80, and 100 mg/mL, cellular viability across all experimental cohorts remained above 90%, exhibiting no statistically significant difference compared to the control group (*p* > 0.05). This strongly indicates that even after prolonged incubation at an extremely high extract concentration (100 mg/mL), the G/3-APBA hydrogel does not inhibit cell proliferation. Live/dead cell staining further corroborated the low toxicity of the gel toward L929 cells. The density of viable cells (green fluorescence) in the hydrogel groups was highly consistent with the control group, with only sparse, sporadic dead cells (red fluorescence) observed. Most fibroblasts maintained their typical spindle-like morphology post-culture (Figure 5C). Quantitative analysis (Figure 5B) confirmed no significant variance in cell survival rates between any of the treatment groups and the control post-24 h. These results compellingly demonstrate that the cells retained robust relative activity post-incubation with the hydrogel, confirming that the material fully satisfies the stringent biosafety requirements for biomedical applications [46].

### 2.5. Antibacterial Performance Evaluation of G/3-APBA

**Figure 6:**
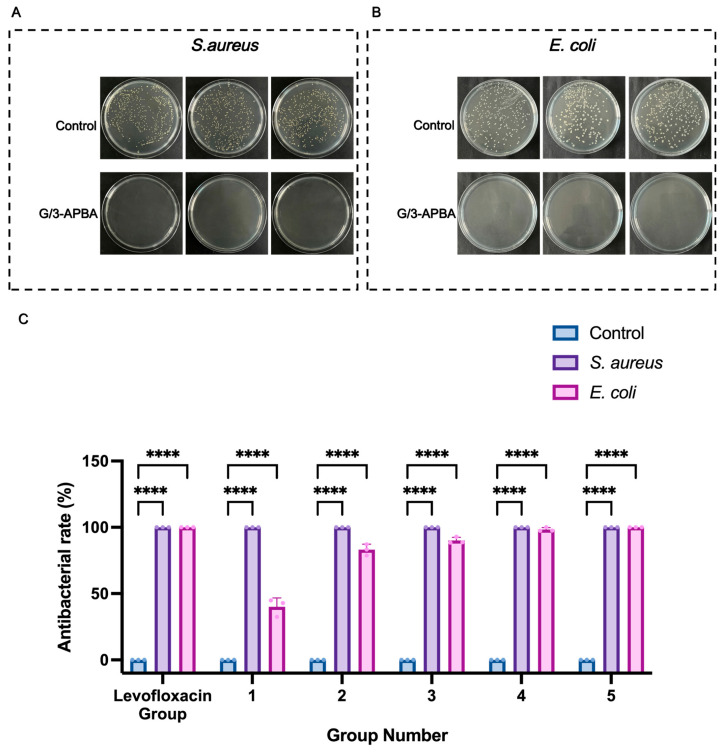
In vitro antibacterial evaluation of the G/3-APBA hydrogel. (**A**) Representative plate-spreading assay images of *S. aureus* after different treatments. (**B**) Representative plate-spreading assay images of *E. coli* after different treatments. (**C**) Quantitative antibacterial rates calculated from CFU counts. Groups 1, 2, 3, 4, and 5 correspond to different masses of G/3-APBA hydrogel, i.e., 0.04, 0.08, 0.12, 0.16, and 0.20 g, respectively. Levofloxacin was used as the positive antibiotic control. Data are presented as the mean ± SD from three independent experiments (*n* = 3, **** *p* < 0.0001).

Guanosine-based supramolecular hydrogels inherently possess dynamic, antibacterial, and peroxidase-like active characteristics, positioning them as highly promising ROS modulation candidates for medical applications, such as chronic wound management [14]. To robustly evaluate the in vitro antibacterial efficacy of the hydrogel, *S. aureus* (Figure 6A) and *E. coli* (Figure 6B) were selected as representative Gram-positive and Gram-negative pathogenic strains, respectively, utilizing the colony-counting method. As demonstrated by the plate spreading assays, an abundance of bacterial colonies proliferated in the control dishes, whereas colony formation was markedly suppressed in the hydrogel-treated groups. Quantitative colony-counting analysis showed that the G/3-APBA hydrogel reduced the number of recoverable viable colonies of both *S. aureus* and *E. coli* under the tested in vitro conditions (Figure 6A,B). For *S. aureus*, the hydrogel-treated groups showed high antibacterial rates across the tested hydrogel mass range. For *E. coli*, the antibacterial rate increased as the hydrogel mass increased from 0.04 to 0.20 g, indicating a hydrogel mass-dependent antibacterial trend (Figure 6C). This mass-dependent response suggests that the reduction in recoverable *E. coli* colonies was not solely caused by nonspecific passive sequestration, but was associated with the amount of hydrogel used during material–bacteria contact. Levofloxacin, used as the positive antibiotic control, markedly reduced colony formation, supporting the validity of the colony-counting assay. Based on the colony-counting results and the structural characteristics of the hydrogel, the possible antibacterial behavior of the G/3-APBA hydrogel may be associated with interactions between bacterial surfaces and the guanosine-derived supramolecular network. The abundant hydrogen-bonding motifs and ordered nucleobase supramolecular assemblies may contribute to interfacial interactions with bacterial surfaces, which could be related to possible membrane-associated antibacterial effects [47]. In addition, the interconnected porous hydrogel network may partially retain bacteria or limit their free diffusion during material–bacteria contact [16,48]. However, to validate the hypotheses for bacterial membrane disruption, contact-killing behavior, or physical entrapment, it still requires further evidence.

## 3. Conclusions

This study successfully engineered an injectable, self-healing hydrogel predicated on G-quadruplex supramolecular self-assembly and 3-aminophenylboronic acid dynamic cross-linking, comprehensively elucidating the influence of formulation parameters on gelation behavior and macroscopic properties. The incorporation of 3-APBA uniquely establishes extensive intermolecular hydrogen bonding and electrostatic stabilization, preventing network collapse under physiological-like conditions. The precise formation of G-quadruplex architectures and dynamic boronate ester cross-links was supported. The optimized hydrogel manifested exceptional macroscopic gelation stability and featured a continuous, interconnected porous network microstructure. Comprehensive rheological profiling revealed that the rigid potassium-stabilized G-quadruplex backbone imparts pronounced shear-thinning behavior, thereby fulfilling the structural prerequisite for low-resistance injectability. Concurrently, the highly reversible nature of the dynamic boronate ester cross-links empowers the matrix with an exceptional 95.3% structural recovery rate following severe mechanical yielding, profoundly justifying its autonomous self-healing capacity in dynamic environments. Biosafety evaluations indicated the hydrogel’s excellent cytocompatibility. Furthermore, biological efficacy assessments revealed that the hydrogel system exerts antibacterial activity under the tested in vitro conditions against critical pathogens, including *S. aureus* and *E. coli*. However, this study is currently limited to in vitro material characterization and preliminary biological evaluation. No in vivo study was performed in the present work. Future studies should further investigate the degradation behavior, retention time at the infected site, inflammatory response, long-term biosafety, and antibacterial efficacy of the G/3-APBA hydrogel in infected wound models. In conclusion, this research provides a highly viable strategic framework for constructing dynamic supramolecular hydrogels that synchronously integrate injectability, self-healing autonomy, and robust preliminary antibacterial efficacy, validating its tremendous translational value as a potential biomaterial and driving its advancement toward multifunctional therapeutic platforms.

## 4. Materials and Methods

### 4.1. Materials

Guanosine (98%) and guanosine 5′-monophosphate disodium salt hydrate (GMP, 98%) were purchased from Energy Chemical Technology Co., Ltd. (Shanghai, China). 3-Aminophenylboronic acid (3-APBA, 98%), 1,4-benzenediboronic acid (BDBA, 97%), and potassium hydroxide (KOH, 95%) were sourced from Macklin Biochemical Co., Ltd. (Shanghai, China). Phenylboronic acid (PBA, 99.5%) and 4-mercaptophenylboronic acid (MPBA, 95%) were purchased from Bidepharm Ltd. (Shanghai, China). Data processing and statistical analyses were performed using Origin 2024 (OriginLab Corporation, Northampton, MA, USA) and GraphPad Prism 10 (GraphPad Software, Boston, MA, USA). Image processing and figure preparation were conducted using Adobe Illustrator 2026 (Adobe Inc., San Jose, CA, USA) and Fiji (ImageJ 1.54t, National Institutes of Health, Bethesda, MD, USA).

### 4.2. Preparation of G/3-APBA Injectable Hydrogel

Guanosine (0.0425 g, 0.15 mmol) was placed into a centrifuge tube (BKMAMLAB, Changde, China), followed by the sequential addition of 1 mL of GMP solution (150 mM), 0.5 mL of KOH solution (150 mM), and 1 mL of ultrapure water. Finally, 0.5 mL of 3-APBA solution (210 mM) was introduced. The optimized molar ratio of guanosine:GMP:3-APBA:KOH is 1:1:0.7:0.5, with final concentrations of 50 mM, 50 mM, 35 mM, and 25 mM, respectively. The mixture was incubated in a 95 °C water bath for approximately 15 min and subsequently cooled to room temperature. Gel formation was confirmed employing the standard vial inversion method. For comparison, 0.5 mL of different concentrations of 3-APBA, BDBA, PBA, and MPBA was used to replace 3-APBA solution (210 mM).

### 4.3. Microstructural Characterization

The fabricated hydrogel samples were rapidly frozen in liquid nitrogen for approximately 10 min. While maintaining the cryogenic environment, the samples were gently fractured to expose the cross-section. The fractured specimens were immediately transferred to a freeze dryer and lyophilized for 6–12 h to ensure complete moisture sublimation. The thoroughly desiccated samples were subsequently sputter-coated. A scanning electron microscope (Ultra Plus, Zeiss, Oberkochen, Germany) was utilized to observe the microscopic morphology and acquire SEM images, facilitating the rigorous analysis of the gel’s pore architecture, wall morphology, and network interconnectivity.

### 4.4. FTIR Analysis

A Fourier Transform Infrared (FTIR) spectrometer (Nicolet iS5, Thermo Fisher Scientific, Waltham, MA, USA) was utilized to meticulously analyze the chemical structure and supramolecular interactions inherent within the hydrogel network. The spectral scanning range was set from 4000 to 400 cm^−1^ with a precise resolution of 4.0 cm^−1^. Both sample and background scans were conducted for 32 accumulations.

### 4.5. Thioflavin T Fluorescence Assay

To indirectly verify the generation of G-quadruplex-related structures within the G/3-APBA hydrogel matrix, Thioflavin T (ThT) was employed as a highly specific fluorescent probe. Thioflavin T (ThT) fluorescence assays were performed to indirectly verify G-quadruplex formation, exploiting the principle of ligand-induced fluorescence enhancement upon ThT binding to G-quadruplex architectures. Maintaining a fixed total volume of 3 mL, the G/3-APBA hydrogel was synthesized via a one-pot method to incorporate a final ThT concentration of 100 μM. The subsequent fluorescence intensity was quantified and benchmarked against a 100 μM aqueous ThT reference solution.

### 4.6. CD Analysis

CD spectra were recorded at room temperature using a circular dichroism spectrometer (J-1500, Jasco Co., Ltd., Hachioji, Japan). The spectra were collected over a wavelength range of 200–350 nm. The corresponding blank was measured under identical conditions and subtracted from the sample spectrum prior to analysis.

### 4.7. Rheological Evaluation

Comprehensive rheological tests were executed utilizing a rotational rheometer (MCR302, Anton Paar, Graz, Austria) at a strictly controlled 37 °C. Freshly formulated gel samples were allowed a structural relaxation period on the measuring plate prior to initialization. Critical frequency sweep tests were performed within a set frequency range of 0.01 Hz to 100 Hz at a constant 1% (low) strain, capturing the dynamic modulus evolution at 10 s intervals. Strain sweep evaluations were conducted through scanning from 0.1% to 1000% strain at a fixed frequency of 1 Hz, and data points were recorded every 10 s to pinpoint the yield threshold. Based on these prior parameters, dynamic step-strain cyclic tests were programmed, alternating between low (1%) and high (500%) strain levels, definitively driving the material through successive gel–sol–gel transitions across 3 complete cycles to accurately quantify structural recovery kinetics.

### 4.8. Biosafety Evaluation

Mouse fibroblast L929 cells (BeNa Culture Collection, Beijing, China), cultured to the logarithmic growth phase, were seeded into a 96-well plate at a density of approximately 5000 cells per well. The cells were maintained in a constant-temperature incubator under standard conditions: 37 °C, near-saturation humidity, and a 5% CO_2_ atmosphere. To prepare the extraction medium, 0.1 g of the G/3-APBA hydrogel was immersed in 10 mL of DMEM supplemented with 15% FBS and incubated in a 37 °C water bath for 12 h. Following cellular adhesion, the media were replaced with varying concentrations (20, 40, 60, 80, and 100 mg/mL) of the prepared hydrogel extraction media. After 24 h of sustained treatment, cell viability was quantified utilizing the established MTT assay to calculate relative cellular survival rates.

Parallelly, L929 cells in logarithmic growth were seeded into a 24-well plate at roughly 10,000 cells per well under identical incubation parameters. Post-adhesion, cells were treated with the designated concentration gradient of hydrogel (20, 40, 60, 80, and 100 mg/mL) extracts for 24 h. A Calcein-AM/PI double-staining protocol was subsequently applied to facilitate a rigorous live/dead visual and quantitative analysis of the fibroblasts co-incubated with the hydrogel.

### 4.9. Antibacterial Performance Evaluation

The antibacterial assays comprised a negative control group, experimental groups treated with different masses of the pristine G/3-APBA hydrogel, and a positive antibiotic control group treated with levofloxacin. *S. aureus* and *E. coli* were utilized as representative pathogenic models, and the standard colony-counting methodology was used to quantify in vitro antibacterial performance. Specifically, 10 μL of bacterial suspension (total density of 1 × 10^6^ CFU/mL) was combined with different masses of the G/3-APBA hydrogel (0.04, 0.08, 0.12, 0.16, and 0.20 g) in sterile shaking tubes and incubated at 37 °C for 2 h to facilitate material–pathogen interaction. For the positive antibiotic control, levofloxacin at a final concentration of 10 μg/mL was added to the bacterial suspension and incubated under the same conditions. After co-incubation, sterile 0.9% saline was added to each tube, followed by vortexing and repeated pipetting to recover bacteria associated with the hydrogel-containing system before serial dilution. The hydrogel-treated bacterial suspensions and control samples were subsequently subjected to 10-fold serial dilutions. Aliquots of 100 μL from the appropriate dilutions were uniformly spread onto LB agar plates and incubated at 37 °C for 12 h. Post-incubation, macroscopic colony formation was documented, and antibacterial rates were calculated.

### 4.10. Statistical Analysis

All quantitative experiments incorporated within this study were performed utilizing a minimum of three independent replicates. Resultant data are systematically expressed as the mean ± standard deviation (mean ± SD). Multi-group data comparisons (e.g., the differential impact of varying hydrogel concentrations on cell viability) were subjected to a one-way analysis of variance (one-way ANOVA), followed by Tukey’s multiple comparison test for rigorous post hoc analysis. A *p*-value < 0.05 was the threshold for statistical significance (ns means *p* > 0.05, * *p* < 0.05, ** *p* < 0.01, and *** *p* < 0.001, **** *p* < 0.0001).

## Data Availability

The dataset is available upon request from the authors.

## Abbreviations

The following abbreviations are used in this manuscript:

3-APBA	3-Aminophenylboronic acid
GMP	Guanosine 5′-monophosphate disodium salt hydrate
G	Guanosine
PBA	Phenylboronic acid
MPBA	4-Mercaptophenylboronic acid
BDBA	1,4-Benzenediboronic acid
KOH	Potassium hydroxide
SEM	Scanning electron microscopy
FTIR	Fourier transform infrared
ThT	Thioflavin T
*S. aureus*	Staphylococcus aureus
*E. coli*	Escherichia coli
SD	Standard deviation
ANOVA	Analysis of variance
